# Genome-wide association study identifies three key loci for high mesocarp oil content in perennial crop oil palm

**DOI:** 10.1038/srep19075

**Published:** 2016-01-08

**Authors:** Chee-Keng Teh, Ai-Ling Ong, Qi-Bin Kwong, Sukganah Apparow, Fook-Tim Chew, Sean Mayes, Mohaimi Mohamed, David Appleton, Harikrishna Kulaveerasingam

**Affiliations:** 1 Biotechnology & Breeding Department, Sime Darby Plantation R&D Centre, Malaysia; 2 Department of Biological Sciences, National University of Singapore, Singapore; 3 School of Biosciences, University of Nottingham, UK

## Abstract

GWAS in out-crossing perennial crops is typically limited by insufficient marker density to account for population diversity and effects of population structure resulting in high false positive rates. The perennial crop oil palm is the most productive oil crop. We performed GWAS for oil-to-dry-mesocarp content (O/DM) on 2,045 genotyped *tenera* palms using 200K SNPs that were selected based on the short-range linkage disequilibrium distance, which is inherent with long breeding cycles and heterogeneous breeding populations. Eighty loci were significantly associated with *O/DM* (*p* ≤ 10^−4^) and three key signals were found. We then evaluated the progeny of a Deli x AVROS breeding trial and a 4% higher O/DM was observed amongst those having the beneficial genotypes at two of the three key loci (*p* < 0.05). We have initiated MAS and large-scale planting of elite *dura* and *pisifera* parents to generate the new commercial *tenera* palms with higher O/DM potential.

Genome-wide association study (GWAS) has emerged as a powerful method and commonly adopted, particularly in human populations to identify a broad range of complex diseases^1^. The method was then wide implemented in annual plants, including rice^2^,^3^,^4^, foxtail millet^5^, maize^6^ and Arabidopsis^7^ when their reference genomes were successfully sequenced. As for perennial crops, GWAS progress is often hindered due to insufficient marker density and population structure effects^8^,^9^. We carried out GWAS on oil palms (*Elaeis guineensis* Jacq.) as a model to address these limiting factors and to illustrate the potential of marker-assisted selection (MAS) in out-crossing breeding programmes. The oil palm with the highest yield per hectare of all oil crops, it accounts for a quarter of vegetable oil traded worldwide annually, despite occupying only 5% of the global oil planting acreage^10^. Only five to six generations of selection and breeding have been completed since plantations were established in the 1920s and 1930s^11^, primarily due to long phenotyping cycles (typically twelve years per cycle).

In recent decades, molecular markers have been employed to identify quantitative trait loci (QTL) for traits of importance in oil palm. By determining the allelic variation present, palms that possess particular combinations of desired QTL can be selected at the nursery stage. Markers could significantly reduce the conventional phenotyping cycles and also enrich the best combinations of alleles in palms planted from each cross. Most marker discovery programmes in oil palm are still mainly based on controlled cross-based linkages using various marker systems with modest density, including restriction fragment length polymorphisms (RFLPs)^12^,^13^, amplified fragment length polymorphisms (AFLPs)^14^ and simple sequence repeats (SSRs)^15^. To further increase marker density, single nucleotide polymorphisms (SNPs) that distribute abundantly throughout a genome, were developed for oil palm^16^. Nevertheless, the application of SNPs was still mainly deployed for linkage map construction^16^ and followed by localisation of fruit form trait with Mendelian inheritance^10^ and stem height trait^17^. While the family-based mapping approach and high-density markers are reasonably powerful for detecting the major QTLs, the mapping resolution, particularly for complex traits, like oil yield is always constrained by small population size. Typically, seed numbers from a single breeding cross exceeds 1,000, but currently only 16–96 palms are randomly selected for assessment.

The publication of the oil palm genome consisting of 1.535 gigabase (Gb) of assembled sequence^10^, the independent assembly of Sime Darby’s oil palm genome and development of high throughput SNP genotyping technologies has enabled us to produce a pipeline for marker-assisted breeding in oil palm, from discovery to the planting of marker-selected breeding materials in the field. We here report the first comprehensive GWAS in oil palm, validation of the results for oil-to-dry mesocarp (O/DM) and the application of the results in a field level selection programme. This is, to our knowledge, the largest SNP array-based GWAS analysis in any tree species and provides a test case for the application of such analysis to address the unique characteristics of perennial food crop species. Such step-wise changes are needed to address the major challenge of food security facing the world.

## Result and Discussion

The Deli *dura* palms are a breeding population of restricted origin (BPRO) derived from the four original palms planted at Bogor Botanical Garden in 1848^18^. Developed in different breeding programmes with different selection objectives, this led to important sub-populations^19^, such as the *Ulu Remis* and *Johore Labis* materials^20^. The Deli origin exhibits thick mesocarp, high bunch number and high O/DM and is the most important oil palm planting material. Shell thickness is inversely related to mesocarp/fruit (hence oil yield) and is controlled by a single gene with two co-dominant alleles, *sh + *and *sh*−21. Planters in Southeast Asia in the 1960s switched from planting Deli *dura* (*sh + sh+*) to planting *tenera* (*sh + sh*−) derived from Deli x AVROS, realizing a 30% increment in oil yield/hectare^22^,^23^. However, this complicated breeding selection by requiring separate development of maternal (*dura*) and paternal (*pisifera*) breeding lines, followed by extensive progeny testing. Concerns of a narrow genetic base limiting future breeding progress has also led many oil palm breeders to evaluate other sources of *dura* germplasm. For this reason, the semi-wild Nigerian *dura* crossed with AVROS *pisifera* was also evaluated in Sime Darby.

**Figure 1:**
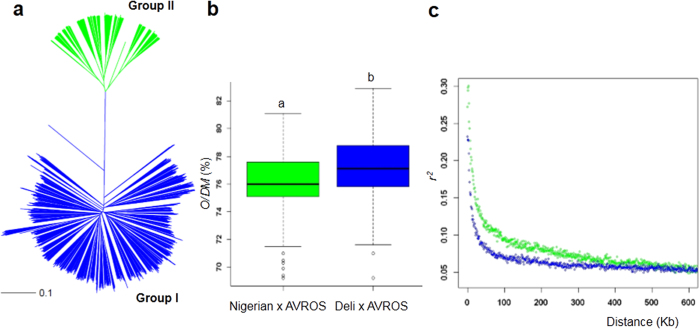
Genetic stratification of 2,045 oil palm samples representing Deli x AVROS and Nigerian x AVROS. (**a**) Neighbor-joining tree (NJ) constructed from Hamming distances for all SNPs. The two divergent groups, Group I and Group II are shown in blue and green, respectively. The scale bar indicates the Hamming distance. (**b**) The boxplots represent median values, percentile 25–75 and outliers for the oil-to-dry mesocarp (O/DM) of the two divergent groups (color as in **a**). Statistical significance for each group was determined by a Student-t test at *p* < 0.001. (**c**) Genome-wide average LD decay rate estimated from 1,459 Deli x AVROS (Group I) and 586 Nigerian x AVROS (Group II) palms (color as in **a**).

One hundred and thirty-two oil palm representing 59 breeding origins (Supplementary Table 1) were sampled for whole genome re-sequencing, yielding ~900 million raw reads. Approximately 60% of the raw reads were mappable to the published oil palm reference genome^10^. We identified 7.8 million potential SNPs, of which 200,000 (with minor allele frequency, MAF >0.05) were selected for array design (see Methods). The array was used to genotype 2,045 palms with existing phenotype data (including oil yield collected over 7 years). A clustering analysis based on Hamming distance (Fig. 1a) showed that the palms could be assigned to two divergent groupings i.e. (I) commercially important Deli x AVROS (n = 1,459) palms and (II) semi-wild Nigerian x AVROS (n = 586) palms. On average, group I yielded 1% O/DM more than the semi-wild group II (*p* < 0.001) (Fig. 1b) (Supplementary Table 2). An increase of 1% in mesocarp oil content (or oil extraction rate, OER) could translate to approximately 500,000 tons of additional oil production annually in Malaysia^24^, without the need of new plantings.

The linkage disequilibrium (LD) decay rates of Deli x AVROS and Nigerian x AVROS were 25Kb and 20Kb at 0.12 and 0.15 of average pairwise correlation coefficients (*r*^*2*^) (Fig. 1c). The LD in the commercially important population derived from a narrow genetic base of Deli founders only decayed slightly slower than the semi-wild population. The long-range LD in Deli *dura* might be broken down by the genetic recombination introduced from AVROS *pisifera* to increase oil yield through heterosis in *tenera*. The LD of the oil palm populations decays considerably faster than in cultivated rice and foxtail millet. In both cereal species, the LD decay ranged from 100Kb to 200Kb^2^,^3^,^4^,^5^ accumulated from a long history of reproductive isolation in self-fertilization breeding systems. Overall, the 200K SNP array with an average sampling of one locus for every 11Kb based on the reference genome size^10^, was selected to provide sufficient genomic resolution for GWAS.

**Figure 2:**
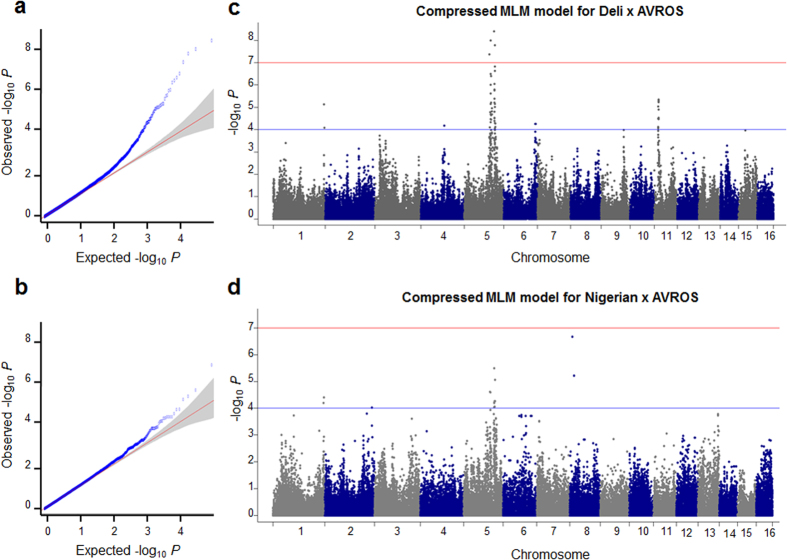
Genome-wide association studies results for oil-to-dry mesocarp (O/DM). (**a**) Quantile-Quantile plot of compressed MLM for Deli x AVROS. (**b**) Quantile-Quantile plot of compressed MLM for Nigerian x AVROS. (**c**) Manhattan plots of Compressed MLM model for Deli x AVROS. Negative log_10_-transformed *p* values from a genome-wide scan are plotted against position on each of the 16 chromosomes. The blue and red horizontal lines indicates the genome-wide significance cut-off and Bonferroni cut-off. (**d**) Manhattan plots of Compressed MLM model for Deli x AVROS, as in **(c)**.

The O/DM of individual palms was measured over 7 years of field planting (summarized in Supplementary Table 2) to perform a phenotype-genotype association analysis using a simple linear model. We discovered inflated false-positive signals (Supplementary Fig. 1) in Deli x AVROS (Genomic inflation factor, GIF = 3.66) and, particularly, in Nigerian x AVROS (GIF = 11.9) (Supplementary Fig. 2). The result suggested that the model failed to account for the recent common ancestry of small groups of individuals, defined as cryptic relatedness^25^,^26^. General linear model (GLM)-based methods, including structure association^27^, genome control^26^ and family-based^28^ tests of association are widely used to address population stratification. We adopted a compressed mixed linear model (MLM) with population parameters previously determined (P3D) to address the problem of genomic inflation that was based on a principle component analysis and a group kinship matrix^29^. This method greatly reduced false positives in Deli x AVROS (GIF = 1.1) and Nigerian x AVROS (GIF = 1.9) as illustrated in Quantile-Quantile plots (Fig. 2a,b).

In total, 62 and 18 significant association signals for the O/DM phenotype (Supplementary Table 3) were mainly clustered on Chromosomes 5 and Chromosome 11 in Deli x AVROS (Fig. 2c) and Nigerian x AVROS (Fig. 2d) guided by a whole-genome significance cutoff (*p*) at 10^−4^ and a Bonferroni cutoff at 10^−7^. The number of significant signals was smaller in latter group, possibly due to smaller population size at only half of Deli x AVROS group. Higher statistical power will be achievable with larger population size. We identified three significant SNPs i.e. SD_SNP_000010418 (*p*_*Deli x AVROS*_ = 2.39 × 10^−6^; *p*_*Nigerian x AVROS*_ = 2.45 × 10^−5^), SD_SNP_000019529 (*p*_*Deli x AVROS*_ = 1.51 × 10^−7^; *p*_*Nigerian x AVROS*_ = 6.13 × 10^−5^) and SD_SNP_000002370 (*p*_*Deli x AVROS*_ = 9.48 × 10^−6^; *p*_*Nigerian x AVROS*_ = 6.39 × 10^−5^) on Chromosome 5, which were commonly present in both groups and situated near gene models (Supplementary Table 4). Compared to the previous controlled-crossed linkage studies^15^,^30^, the significant QTLs for O/DM were located on Chromosome 3 and Chromosome 6 in multi-parent progeny from Deli *dura* x Yangambi/La Mé *pisifera* (299 palms) and a progeny from Topi Deli *dura* x Yangambi *pisifera* (69 palms). Interestingly, we also observed one of the QTLs located on Chromosome 6 between 41,545,028bp–41,557,789bp (Supplementary Table 3). The major association peak on Chromosome 5 as reported in this study was however not previously observed. We further analyzed the homologies of candidate genes in or close to the three significant SNPs, which might be influencing the phenotype, using known comparative gene and pathway analyses in oil palm and date palm^31^. For example, SD_SNP_000002370 (physical position: 40,396,733bp; Chromosome 5) located at 2-Kb away from the *Pyruvate kinase* (*PK*) gene was found to be associated to O/DM. The expression of *PK* gene in oil palm was reported to be responsible for an increased flux through to pyruvate for fatty acid biosynthesis^31^,^32^ leading to more oil. Nevertheless, we are currently further fine mapping these loci with higher SNP density.

**Figure 3:**
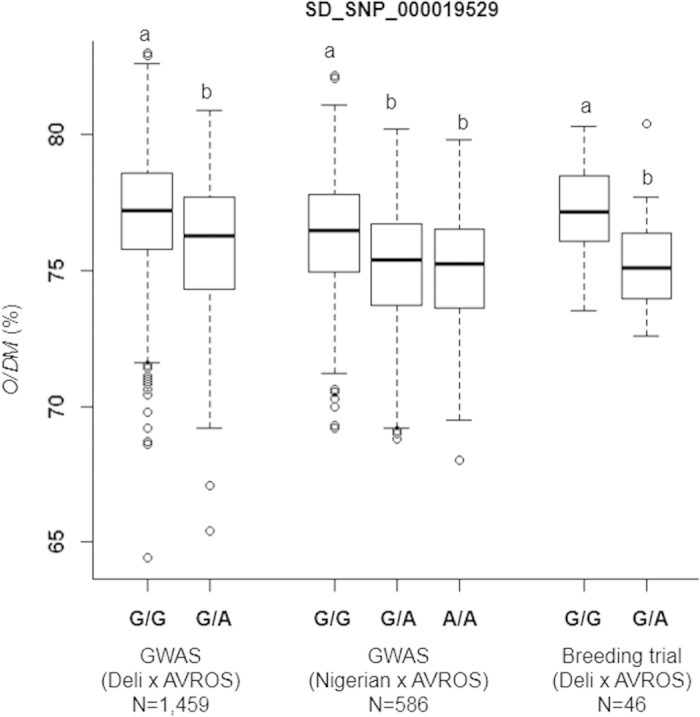
Boxplots of the oil-to-dry-mesocarp (O/DM) trait, grouped according to SNP polymorphism revealed by SD_SNP_000019529. Statistical significance for each genotype in GWAS discovery populations i.e. Deli x AVROS and Nigerian × AVROS was determined by a compressed MLM model at *p* = 1.51 × 10^−7^ and *p* = 6.13 × 10^−5^, respectively. Statistical significance for differences between genotypes in the Deli x AVROS breeding trial was determined by one-way ANOVA at *p* < 0.005.

Oil palm breeders deploy a variant of reciprocal recurrent selection (RRS) or family and individual selection (FIS), producing thick-shelled *dura* maternal and shell-less *pisifera* paternal pools^23^. Elite *dura* and *pisifera* with good combining ability evaluated via progeny testing are crossed for thin-shelled *tenera* commercial production. Within the Deli x AVROS group (1,459 palms), the homozygous G/G palms for SD_SNP_000019529 yielded a mean of 77.1% O/DM, which was significantly higher than the 75.8% observed amongst the heterozygous G/A palms (Fig. 3). The same SNP effect was observed in the Nigerian x AVROS group (586 palms) (O/DM: G/G = 76.3%, G/A = 75.1%, A/A = 74.9%) and further, was successfully validated in a small independent Deli x AVROS breeding trial (46 palms) (Fig. 3). The validation of SD_SNP_000002370 was also proven using the same approach (Supplementary Fig. 3). However, SD_SNP_000010418 did not significantly explain the phenotype variation in the breeding trial, possibly due to insufficient statistical power (Supplementary Fig. 4). In addition, we also observed additive effect when combining the three important SNPs in both groups. The O/DM yield significantly increased along with the stacking of positive alleles in individual palms (Supplementary Fig. 5 and Supplementary Fig. 6).

**Figure 4:**
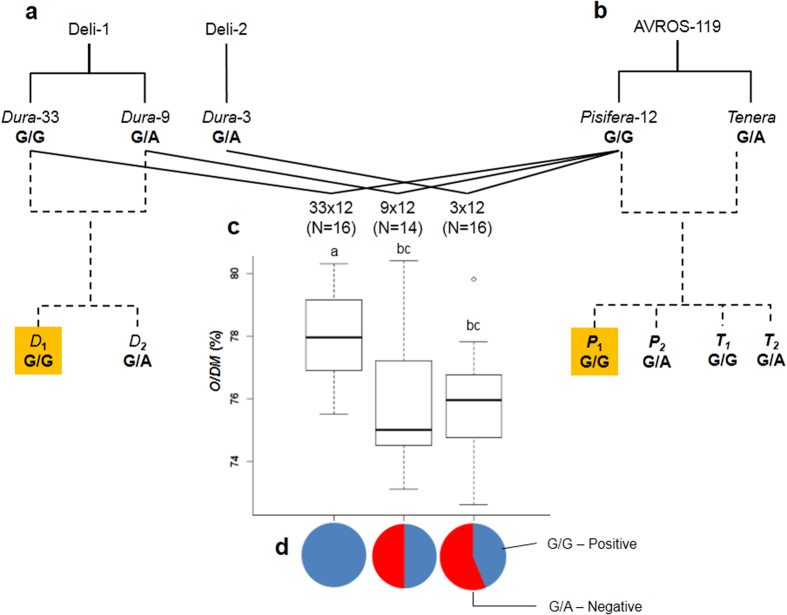
Marker-assisted selection (MAS) of Deli *dura* palms using SD_SNP_000019529. (**a**) The pedigree of Deli *dura* with known genotypes. A new Deli population which consists of G/G (*D*_*1*_) and G/A (*D*_*2*_) genotypes was generated from *Dura-33* x *Dura*-9. Only *D*_*1*_palms (highlighted in orange color) were field planted. *D*–*dura* palm. (**b**) The pedigree of AVROS-199 with known genotypes. A new AVROS population which consists of *G/G* (*P*_*1*_ and *T*_*1*_) and G/A (*P*_*2*_ and *T*_*2*_). Only *P*_*1*_ palms (highlighted in orange color) were field planted. *P*–*pisifera* palm and *T*–*tenera* palm. (**c**) The boxplots represent median values, percentile 25–75 and outliers for the oil-to-dry mesocarp trait (O/DM) of the three progeny testing populations (*Tenera*), derived from the pedigrees shown in a and b. (**d**) The genotype composition for each progeny testing population. Red and blue indicates G/A as negative genotype and G/G as positive genotype for O/DM, respectively.

The most significant locus, SD_SNP_000019529 was selected to illustrate the marker application in an oil palm breeding programme. In the Deli x AVROS breeding trial (Fig. 4), the best combining ability of the *Dura*-33 and *Pisifera*-12 cross (Fig. 4a,b) was associated with the parent carrying the homozygous G/G SD_SNP_000019529; thus, each parent contributed the positive G allele to form a 100% homozygous G/G *tener*a progeny (*Dura* -33 x *Pisifera*-12; n = 16). The O/DM mean for this progeny was 4% higher than the other two progeny (*p* < 0.005; one-way Analysis of Variance) (Fig. 4c), while the poorer combining ability of the remaining *dura* parents was due to transmission of the A alleles (Fig. 4d). Based on these results, we implemented a MAS programme through screening of large number of seedlings for Deli *dura* and AVROS *pisifera* parents derived from *Dura-*33 x *Dura-*9 and *Pisifera-12* x *Tenera*, respectively, using SD_SNP_000019529. Only the homozygous G/G *dura* and *pisifera* seedlings were planted.

In summary, we report the most comprehensive use of high density SNP genotyping in oil palm to date, the use of a GWAS approach to identify SNP variants associated with differences in the key oil-to-dry mesocarp yield trait, and confirmation of their action in an independent cross. Based on these results, we have implemented a MAS programme to breed new parental lines for commercial oil palm hybrid production. The reported study also lays the foundations for a genomic selection model for oil palm and will act as a model for other perennial tree crops.

## Methods

### Sampling and DNA preparation

For re-sequencing, we sampled 132 palms belonging to 59 origins (Supplementary Table 1) maintained at the Sime Darby Plantation R&D Centre in Malaysia. The sampling was then extended to the GWAS discovery populations derived from Deli x AVROS (1,459 palms) and Nigerian x AVROS (586 palms).The sample selection was based on progeny/BPRO of relevance to the breeding programme, followed by phenotypic data availability. After a field census, we selected 5–15 palms from each progeny. For validation, we identified a nested-mating designed trial of Deli x AVROS that was generated previously to determine the combining ability of different *dura* palms to a common *Pisifera-12* (Fig. 4a,b). Three progeny testing populations (46 *tenera*) with their parents (3 Deli *dura*, 1 AVROS *pisifera* and 1 AVROS *tenera*) were sampled. Total genomic DNA was isolated from young leaf tissue (frond 0) using the DNAeasy Plant Mini Kit (Qiagen).

### Whole-genome re-sequencing and genotyping

The 132 samples were pooled based on an equal molar concentration of DNA from each sample to form the sequencing DNA pool. A library was prepared for re-sequencing using Illumina HiSeq 2000 to generate 100-bp pair-end reads to give a 35x genome coverage from 924,271,650 raw reads. The pair-end reads were trimmed, filtered and aligned to the published oil palm genome^10^ using BWA Mapper^33^ with default parameters. A total of 7,755,949 putative SNPs were then called and filtered using SAMtools^34^, with parameters of the minimal mapping quality score of the SNP being 25, minimal depth 3x, and minimal SNP distance from a gap of 2bp. We removed 1,085,204 SNPs that were generated from *E. oleifera* and 746,092 SNPs based on coverage (minimal 17 or maximal 53), genotype quality with minimal score of 8 and minor allele frequency (MAF < 0.05). The other filtering step removed 5,330,765 SNPs based on the technical requirements of Illumina, including the removal of pairs of SNPs with distance less than 60bp apart, ambiguous nucleotides, indels, non-biallelic and A/T or C/G types. We identified 593,888 quality SNPs. According to linkage disequilibrium with a *r*^*2*^ cut-off set at 0.3, 200,000 SNPs with an average density of one SNP per 11Kb were submitted to Illumina for design score calculation using Illumina’s Assay Design Tool for Infinium. The Infinium array, termed as OP200K was used to assay the GWAS discovery populations (~250 ng DNA/sample). The overnight amplified DNA samples were then fragmented by a controlled enzymatic process that did not require gel electrophoresis. The re-suspended DNA samples were hybridized to the BeadChips after an overnight incubation in the capillary flow-through chamber. The allele specific hybridizations were fluorescently labeled and detected by an Illumina BeadArray Reader. The raw reads were then analyzed using the GenomeStudio Data Analysis software for automated genotyping calling and quality control. To generate the genotypic dataset for GWAS, only the SNPs that had minor allele frequency (MAF) >0.01 and >90% call rate were accepted. The missing genotype of accepted SNPs was subsequently imputed based on the mean of each marker^35^.

### Genetic stratification and population analyses

A Neighbor-Joining (NJ) tree was used to infer the genetic stratification of the GWAS discovery populations. A Hamming’s pairwise distance matrix for all SNP sites was calculated to plot the NJ tree. The genome-wide LD decay rates in the Deli x AVROS and Nigerian x AVROS were important to anticipate the requirements for the suitable mapping resolution of the SNP array for GWAS. The rate is defined as the chromosomal distance at which the average pairwise correlation coefficient (*r*^*2*^) dropped to the half of its maximum value. In this study, we calculated the pairwise *r*^*2*^ for all SNPs in a 1-Kb window and averaged across the whole genome based on the composite method in the R package SNPRelate^36^.

### Phenotypic data compilation and genome wide association study (GWAS)

Oil-to-dry-mesocarp (O/DM) is a direct measurement of crude palm oil (CPO) extracted from dry mesocarp tissue using a solvent. The individual palms were phenotyped to produce a reliable mean O/DM value for analysis as per standard industry practice^37^ with modifications^38^. The O/DM difference between Deli x AVROS and Nigerian x AVROS groups was tested for significance by a Student-t test. Subsequently, association analysis was conducted on 1,459 Deli x AVROS and 586 Nigerian x AVROS, respectively, based on a simple linear model in the R package GenABEL^39^, and a compressed MLM with P3D analysis^29^ in the rrBLUP^35^ programme. The total number of common SNPs in both groups was 55,054 SNPs with MAF >0.01. We accounted for the genetic sub-structure resulting from cryptic relatedness by including a kinship matrix^40^ as a random effect in the compressed MLM method. The whole-genome significance cut-offs were fixed at *p* ≤ 10^−4^, and ≤10^−7^, based on a Bonferroni correction method. The Quantile-Quantile plots and Manhattan plots were then constructed using the R package qqman^41^. We also evaluated the inflated false-positive signals for both methods according to the genomic inflation factor (GIF) estimated in R package GenABEL^39^.

### SNP effect and statistical analyses

The common association signals in both groups based on *p* ≤ 10^−4^ were selected to measure the SNP effects on O/DM phenotype and further validated in a Deli x AVROS breeding trial separately. One-way ANOVA tests with multiple comparisons were performed to test the SNP genotypes against the O/DM trait variation. The same approach was applied to determine the SNP effects of the combination of significant SNPs based on the number of positive alleles carried by individual palms. All the statistical analyses were implemented in Minitab 14^42^. The genotype composition in pie charts were also incorporated into a boxplot of the SNP effects on O/DM variation across progeny testing populations to evaluate the combining abilities between *dura* palms and the common *pisifera*. The profiled SNP effects were subsequently used to implement MAS on newly propagated *dura* and *pisifera* seedlings at the pre-nursery stage and followed by field planting.

### Data availability

All the SNPs used in the OP200K genotyping array have been deposited in dbSNP under the handle of SDTC_BB with NCBI submitted SNP (ss) accession numbers of 181006940–1810592638.

## Additional Information

**How to cite this article**: Teh, C.-K. *et al.* Genome-wide association study identifies three key loci for high mesocarp oil content in perennial crop oil palm. *Sci. Rep.* **6**, 19075; doi: 10.1038/srep19075 (2016).

## Supplementary Material

Supplementary Information
