# Supplementary material for: Genome-wide association study identifies three key loci for high mesocarp oil content in perennial crop oil palm
Source: Sci Rep. 2016 Jan 8;6:19075. doi: 10.1038/srep19075 (PMC4705476; doi:10.1038/srep19075)
Supplement: Supplementary Information [file srep19075-s1.doc]

**Genome-wide association study identifies three key loci for high mesocarp oil content in perennial crop oil palm**

Chee-Keng Teh^1^, Ai-Ling Ong^1^, Qi-Bin Kwong^1^, Sukganah Apparrow^1^, Fook-Tim Chew^2^, Sean Mayes^3^, Mohaimi Mahamed^1^, David Appleton^1^ & Harikrishna Kulaveerasingam^1^

^1^Biotechnology & Breeding Department, Sime Darby Plantation R&D Centre, Malaysia. ^2^Department of Biological Sciences, National University of Singapore, Singapore. ^3^School of Biosciences, University of Nottingham, UK.

Correspondence should be addressed to: C.K.T. (teh.chee.keng@simedarby.com)

**Supplementary Materials**

**Index**

**Supplementary Figure 1. Quartile-Quartile (Q-Q) plot of the observed *p* values for GWAS based on simple linear model in Deli x AVROS.**

**Supplementary Figure 2. Quartile-Quartile (Q-Q) plot of the observed *p* values for GWAS based on simple linear model in Nigerian x AVROS.**

**Supplementary Figure 3. Boxplot of SNP polymorphism and SNP effects of SD_SNP_000002370 on the oil-to-dry mesocarp (O/DM) phenotype in Deli x AVROS group, Nigerian x AVROS group and Deli x AVROS breeding trial.**

**Supplementary Figure 4. Boxplot of SNP polymorphism and SNP effects of SD_SNP_000010418 on the oil-to-dry mesocarp (O/DM) phenotype in Deli x AVROS group, Nigerian x AVROS group and Deli x AVROS breeding trial.**

**Supplementary Figure 5. Boxplot of SNP effects of the combination of SD_SNP_000010418, SD_SNP_000019529 and SD_SNP_000002370 on the oil-to-dry mesocarp (O/DM) phenotype in Deli x AVROS group.**

**Supplementary Figure 6. Boxplot of SNP effects of the combination of SD_SNP_000010418, SD_SNP_000019529 and SD_SNP_000002370 on the oil-to-dry mesocarp (O/DM) phenotype in Nigerian x AVROS group.**

**Supplementary Table 1. The 59 origins of the 132 re-sequenced oil palms.**

**Supplementary Table 2. The distribution of oil-to-dry mesocarp (O/DM) for Deli x AVROS (Group I) and Nigerian x AVROS (Group II).**

**Supplementary Table 3. Summary of association results for 77 SNPs in Deli x AVROS and Nigerian x AVROS.**

**Supplementary Table 4. Summary of association results for SNPs in Deli x AVROS and Negerian x AVROS.**


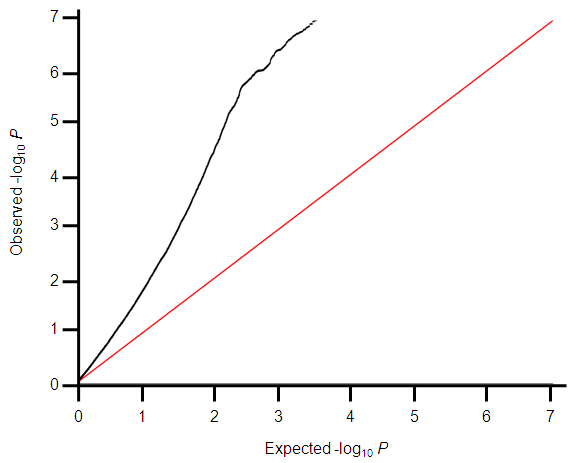


**Supplementary Figure 1. Quartile-Quartile (Q-Q) plot of the observed *p* values for GWAS based on simple linear model in Deli x AVROS.**

The red line is for null expectation; the plot in black is for the *p* values from all the 55,054 SNPs


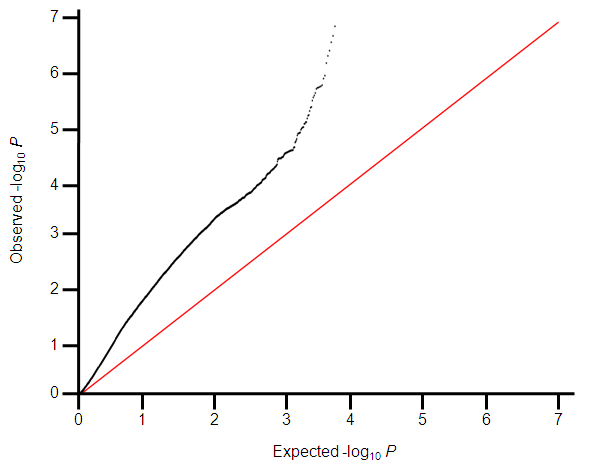


**Supplementary Figure 2. Quartile-Quartile (Q-Q) plot of the observed *p* values for GWAS based on simple linear model in Nigerian x AVROS.**

The red line is for null expectation; the plot in black is for the *p* values from all the 55,054 SNPs


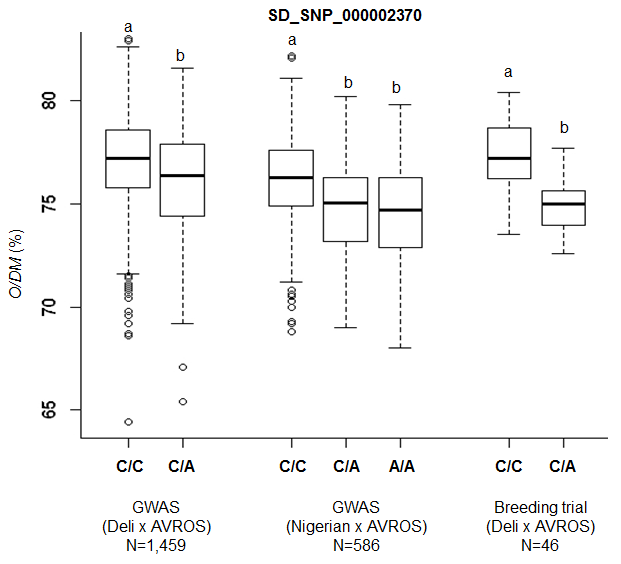


**Supplementary Figure 3. Boxplot of SNP polymorphism and SNP effects of SD_SNP_000002370 on the oil-to-dry mesocarp (O/DM) phenotype in Deli x AVROS group, Nigerian x AVROS group and Deli x AVROS breeding trial.**

Statistical significance for each genotype in GWAS discovery populations i.e. Deli x AVROS and Nigerian x AVROS was determined by compressed MLM model at *p*=9.48 x 10^-6^ and *p*=6.39 x 10^-5^, respectively.

Statistical significance for each genotype class in the Deli x AVROS breeding trial was determined by a one-way ANOVA at *p*<0.005.


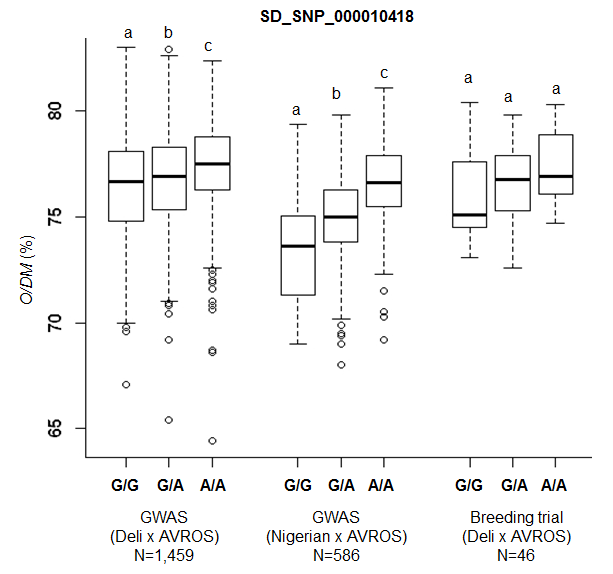


**Supplementary Figure 4. Boxplot of SNP polymorphism and SNP effects of SD_SNP_000010418 on the oil-to-dry mesocarp (O/DM) phenotype in Deli x AVROS group, Nigerian x AVROS group and Deli x AVROS breeding trial.**

Statistical significance for each genotype in GWAS discovery populations i.e. Deli x AVROS and Nigerian x AVROS was determined by compressed MLM model at *p*=2.39 x 10^-6^ and *p*=2.45 x 10^-5^, respectively.

Statistical significance between each genotype class in the Deli x AVROS breeding trial was determined by a one-way ANOVA at *p*<0.005.


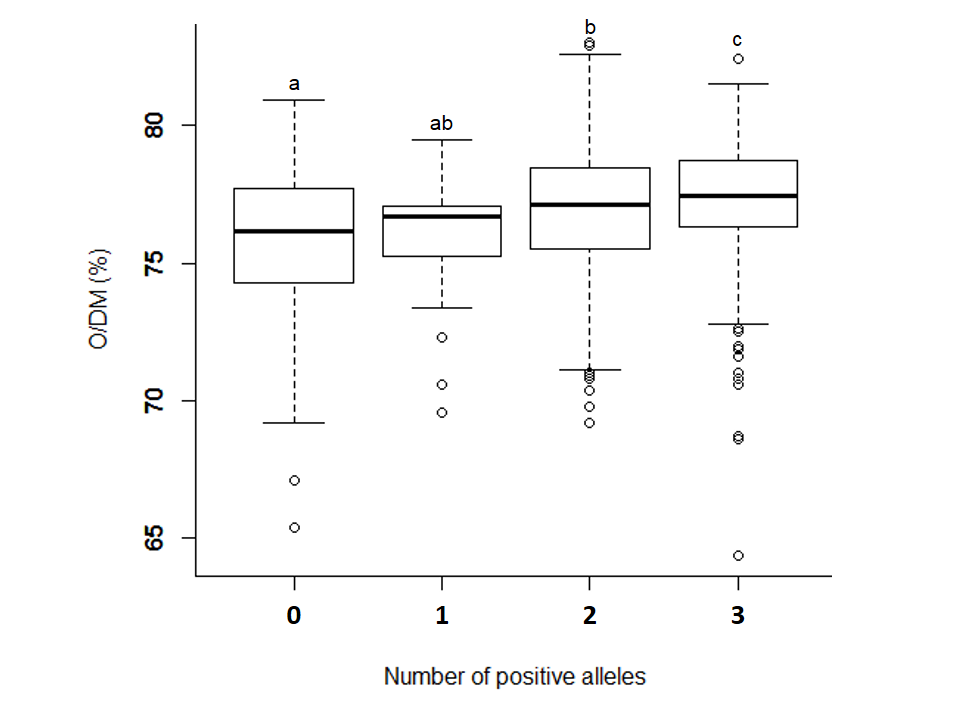


**Supplementary Figure 5. Boxplot of SNP effects of the combination of SD_SNP_000010418, SD_SNP_000019529 and SD_SNP_000002370 on the oil-to-dry mesocarp (O/DM) phenotype in Deli x AVROS group.**

The positive alleles were defined based on the SNP effects of each single significant SNP (A/A for SD_SNP_000010418; G/G for SD_SNP_000019529; C/C for SD_SNP_000002370).

Statistical significance between each number of positive alleles in the Deli x AVROS group was determined by a one-way ANOVA at *p*<0.005.


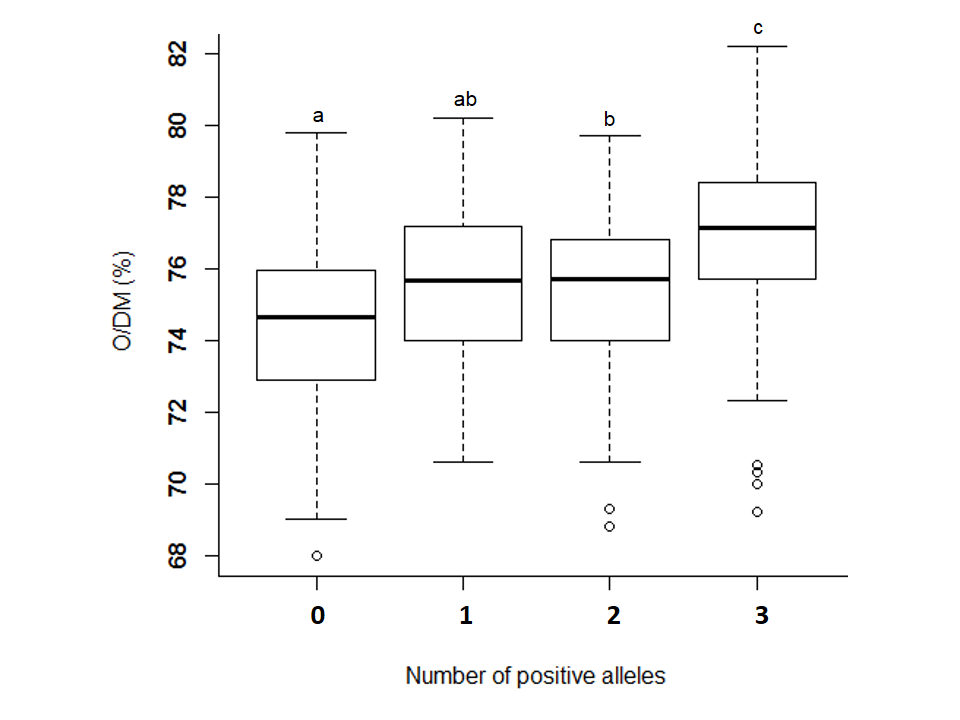


**Supplementary Figure 6. Boxplot of SNP effects of the combination of SD_SNP_000010418, SD_SNP_000019529 and SD_SNP_000002370 on the oil-to-dry mesocarp (O/DM) phenotype in Nigerian x AVROS group.**

The positive alleles were defined based on the SNP effects of each single significant SNP (A/A for SD_SNP_000010418; G/G for SD_SNP_000019529; C/C for SD_SNP_000002370).

Statistical significance between each number of positive alleles in the Nigerian x AVROS group was determined by a one-way ANOVA at *p*<0.005.

**Supplementary Table 1. The 59 origins of the 132** re-sequenced oil palms

| **No.** | **Origin** | **Materials** |
| --- | --- | --- |
| 1 | *Algemene Vereniging van Rubberplanters ter Oostkust van Sumatra (AVROS) 1* | 1 |
| 2 | *AVROS 2* | 1 |
| 3 | *Banting Dura (BD)* | 1 |
| 4 | *Gunung Melayu (GM)* | 1 |
| 5 | *Highland Research Unit (HRU) 1* | 1 |
| 6 | *HRU 2* | 1 |
| 7 | *HRU 3* | 1 |
| 8 | *HRU 4* | 1 |
| 9 | *Johore Labis (JL)* | 1 |
| 10 | *Ulu Remis (UR)* | 1 |
| 11 | *Ulu Remis Tenera (URT) 1* | 1 |
| 12 | *URT 2* | 1 |
| 13 | *URT 3* | 1 |
| 14 | *URT 4* | 1 |
| 15 | *AVROS x Institut pour Researche sur les Huiles et Oléagineux (IRHO)* | 1 |
| 16 | *(BD x Nigerian Institute for Oil Palm Research (NIFOR)) x Jenderata* | 1 |
| 17 | *Deli x AVROS* | 1 |
| 18 | *Deli x Ekona* | 1 |
| 19 | *(E.* guineensis x *E. oleifera) hybrid x AVROS* | 1 |
| 20 | *Ekona x AVROS* | 1 |
| 21 | *GM x Dumpy AVROS* | 1 |
| 22 | *JL x AVROS* | 1 |
| 23 | *JL x Dumpy AVROS* | 1 |
| 24 | *JL x HRU* | 1 |
| 25 | *JL x IRHO* | 1 |
| 26 | *(JL x HRU) x AVROS* | 1 |
| 27 | *NIFOR x AVROS* | 1 |
| 28 | *(NIFOR x DA)1* | 1 |
| 29 | *(NIFOR x DA)2* | 1 |
| 30 | *NIFOR X IRHO* | 1 |
| 31 | *Nigerian x AVROS* | 1 |
| 32 | *Serdang Avenue x AVROS* | 1 |
| 33 | *UR x AVROS* | 1 |
| 34 | *UR x Dumpy AVROS* | 1 |
| 35 | *UR x IRHO* | 1 |
| 36 | *UR x Lobe* | 1 |
| 37 | *(UR x NIFOR)1* | 1 |
| 38 | *(UR x NIFOR)2* | 1 |
| 39 | *(UR x NIFOR)3* | 1 |
| 40 | *UR x Serdang AVROS* | 1 |
| 41 | *UR x Serdang pisifera* | 1 |
| 42 | *UR x URT* | 1 |
| 43 | *(URT x AVROS)1* | 1 |
| 44 | *(URT x AVROS)2* | 1 |
| 45 | *(URT x AVROS)3* | 1 |
| 46 | *(URT x AVROS)4* | 1 |
| 47 | *(URT x AVROS)5* | 1 |
| 48 | *URT x Cameroon* | 1 |
| 49 | *Cameroon 1* | 2 |
| 50 | *Cameroon 2* | 2 |
| 51 | *Nigerian 1* | 2 |
| 52 | *Nigerian 2* | 2 |
| 53 | *Nigerian 3* | 2 |
| 54 | *Tanzanian* | 2 |
| 55 | *E. oleifera 1* | 3 |
| 56 | *E. oleifera 2* | 3 |
| 57 | *E. oleifera 3* | 3 |
| 58 | *E. oleifera 4* | 3 |
| 59 | *E. oleifera 5* | 3 |

Note: 1 – Breeding; 2 – Germplasm & 3 – relative species, *E. oleifera*

**Supplementary Table 2. The distribution of oil-to-dry mesocarp (O/DM) for Deli x AVROS (Group I) and Nigerian x AVROS (Group II).**

| **Group** | **Sample size** | ***O/DM*** | **CV** | ***p*** |
| --- | --- | --- | --- | --- |
| I | 1,458 | 76.88 (±2.29) | 2.97 | < 0.001 |
| II | 584 | 75.67 (±2.43) | 3.22 |

CV - Coefficient of variation; the *p* value was calculated by a one-way ANOVA test.

**Supplementary Table 3. Summary of association results for 77 SNPs in Deli x AVROS and Nigerian x AVROS.**

| SNP ID | INSDC | Chromosome | Position (bp) | Deli x AVROS | | | | Nigerian x AVROS | | | |
| --- | --- | --- | --- | --- | --- | --- | --- | --- | --- | --- | --- |
|  |  |  |  | Major allele | Minor allele | MAF | *p* value | Major allele | Minor allele | MAF | *p* value |
| SD_SNP_000002127 | CM002081.1 | 1 | 66,639,699 | A | G | 0.0576 | *7.32 x 10^-6^ | A | G | 0.352 | 4.01 x 10^-1^ |
| SD_SNP_000016244 | CM002081.1 | 1 | 66,972,538 | G | A | 0.4599 | 8.07 x 10^-1^ | A | G | 0.386 | *6.36 x 10^-5^ |
| SD_SNP_000013063 | CM002081.1 | 1 | 67,033,874 | A | G | 0.0627 | *8.42 x 10^-5^ | A | G | 0.307 | 1.16 x 10^-1^ |
| SD_SNP_000049433 | CM002081.1 | 1 | 67,248,054 | A | C | 0.1309 | 8.11 x 10^-1^ | A | C | 0.234 | *3.94 x 10^-5^ |
| SD_SNP_000038645 | CM002082.1 | 2 | 62,287,970 | G | A | 0.0007 | 1.00 x 10^+0^ | G | A | 0.123 | *9.49 x 10^-5^ |
| SD_SNP_000006192 | CM002084.1 | 4 | 31,149,521 | G | A | 0.1228 | *6.54 x 10^-5^ | G | A | 0.112 | 5.40 x 10^-1^ |
| SD_SNP_000049049 | CM002085.1 | 5 | 32,866,989 | A | G | 0.0691 | *4.22 x 10^-8^ | A | G | 0.242 | 9.33 x 10^-1^ |
| SD_SNP_000039298 | CM002085.1 | 5 | 33,457,617 | A | G | 0.3855 | *7.84 x 10^-5^ | G | A | 0.354 | 7.06 x 10^-1^ |
| SD_SNP_000016161 | CM002085.1 | 5 | 33,975,929 | G | A | 0.0799 | *1.64 x 10^-5^ | G | A | 0.292 | 1.77 x 10^-1^ |
| SD_SNP_000003832 | CM002085.1 | 5 | 34,454,937 | G | A | 0.3909 | *4.68 x 10^-5^ | G | A | 0.487 | 2.60 x 10^-1^ |
| SD_SNP_000018373 | CM002085.1 | 5 | 34,521,370 | G | A | 0.1366 | *3.34 x 10^-5^ | G | A | 0.32 | 1.37 x 10^-1^ |
| SD_SNP_000018372 | CM002085.1 | 5 | 34,525,454 | G | A | 0.1486 | *9.70 x 10^-7^ | G | A | 0.422 | 2.65 x 10^-1^ |
| SD_SNP_000037422 | CM002085.1 | 5 | 34,603,394 | G | A | 0.3718 | *7.09 x 10^-6^ | G | A | 0.384 | 6.93 x 10^-3^ |
| SD_SNP_000040073 | CM002085.1 | 5 | 34,612,126 | G | A | 0.0741 | *3.20 x 10^-7^ | G | A | 0.176 | 8.66 x 10^-4^ |
| SD_SNP_000022444 | CM002085.1 | 5 | 34,773,282 | A | G | 0.145 | *2.11 x 10^-5^ | A | G | 0.389 | 7.83 x 10^-1^ |
| SD_SNP_000010418 | CM002085.1 | 5 | 34,828,628 | G | A | 0.3796 | *2.39 x 10^-6^ | G | A | 0.247 | *2.45 x 10^-5^ |
| SD_SNP_000015218 | CM002085.1 | 5 | 34,856,258 | C | A | 0.1623 | *1.02 x 10^-8^ | C | A | 0.327 | 1.28 x 10^-3^ |
| SD_SNP_000015219 | CM002085.1 | 5 | 34,863,191 | A | G | 0.3838 | *2.28 x 10^-5^ | A | G | 0.3 | 4.15 x 10^-3^ |
| SD_SNP_000042931 | CM002085.1 | 5 | 34,980,654 | G | A | 0.3848 | *6.07 x 10^-6^ | A | G | 0.356 | 1.14 x 10^-4^ |
| SD_SNP_000048207 | CM002085.1 | 5 | 35,080,572 | A | G | 0.2922 | *3.51 x 10^-5^ | G | A | 0.196 | 7.21 x 10^-1^ |
| SD_SNP_000024668 | CM002085.1 | 5 | 35,100,527 | A | G | 0.0761 | *4.02 x 10^-7^ | A | G | 0.32 | 4.57 x 10^-2^ |
| SD_SNP_000024664 | CM002085.1 | 5 | 35,121,695 | A | G | 0.0918 | *2.29 x 10^-5^ | A | G | 0.298 | 2.65 x 10^-3^ |
| SD_SNP_000050827 | CM002085.1 | 5 | 35,192,678 | A | G | 0.3113 | 1.75 x 10^-1^ | A | G | 0.248 | *2.54 x 10^-5^ |
| SD_SNP_000033957 | CM002085.1 | 5 | 36,158,880 | G | A | 0.0689 | *1.10 x 10^-5^ | G | A | 0.445 | 4.51 x 10^-1^ |
| SD_SNP_000030440 | CM002085.1 | 5 | 36,218,554 | A | G | 0.074 | *2.07 x 10^-5^ | A | G | 0.333 | 1.40 x 10^-1^ |
| SD_SNP_000030409 | CM002085.1 | 5 | 36,234,729 | G | A | 0.0773 | *1.52 x 10^-5^ | G | A | 0.394 | 1.63 x 10^-1^ |
| SD_SNP_000024845 | CM002085.1 | 5 | 39,210,662 | A | G | 0.0668 | *3.89 x 10^-9^ | A | G | 0.166 | 1.39 x 10^-3^ |
| SD_SNP_000054111 | CM002085.1 | 5 | 39,607,208 | G | A | 0.2975 | *6.15 x 10^-6^ | A | G | 0.379 | 6.62 x 10^-1^ |
| SD_SNP_000054110 | CM002085.1 | 5 | 39,610,847 | A | G | 0.2968 | *1.56 x 10^-5^ | G | A | 0.379 | 6.62 x 10^-1^ |
| SD_SNP_000054109 | CM002085.1 | 5 | 39,613,906 | A | G | 0.29 | *4.17 x 10^-6^ | G | A | 0.478 | 5.73 x 10^-1^ |
| SD_SNP_000054992 | CM002085.1 | 5 | 39,620,126 | G | A | 0.29 | *4.17 x 10^-6^ | A | G | 0.478 | 5.73 x 10^-1^ |
| SD_SNP_000054080 | CM002085.1 | 5 | 39,642,505 | A | C | 0.2973 | *6.16 x 10^-6^ | C | A | 0.378 | 6.44 x 10^-1^ |
| SD_SNP_000053315 | CM002085.1 | 5 | 39,653,455 | G | A | 0.3873 | *1.58 x 10^-6^ | G | A | 0.444 | 1.41 x 10^-2^ |
| SD_SNP_000051833 | CM002085.1 | 5 | 39,763,460 | C | A | 0.3807 | *1.77 x 10^-6^ | A | C | 0.491 | 1.64 x 10^-4^ |
| SD_SNP_000047120 | CM002085.1 | 5 | 39,799,450 | G | A | 0.4225 | *2.99 x 10^-5^ | A | G | 0.489 | 7.82 x 10^-3^ |
| SD_SNP_000047117 | CM002085.1 | 5 | 39,804,720 | A | G | 0.4218 | *3.97 x 10^-5^ | G | A | 0.489 | 7.82 x 10^-3^ |
| SD_SNP_000046882 | CM002085.1 | 5 | 39,805,514 | A | G | 0.4217 | *2.97 x 10^-5^ | G | A | 0.489 | 7.82 x 10^-3^ |
| SD_SNP_000047116 | CM002085.1 | 5 | 39,806,797 | G | A | 0.422 | *3.07 x 10^-5^ | A | G | 0.417 | 2.09 x 10^-2^ |
| SD_SNP_000048815 | CM002085.1 | 5 | 39,860,983 | G | A | 0.3737 | *9.14 x 10^-7^ | G | A | 0.451 | 3.55 x 10^-4^ |
| SD_SNP_000014128 | CM002085.1 | 5 | 39,966,907 | A | G | 0.1305 | *7.07 x 10^-6^ | A | G | 0.34 | 2.52 x 10^-3^ |
| SD_SNP_000019028 | CM002085.1 | 5 | 40,066,844 | A | G | 0.1757 | *2.35 x 10^-7^ | G | A | 0.493 | 9.71 x 10^-4^ |
| SD_SNP_000022774 | CM002085.1 | 5 | 40,112,108 | G | A | 0.4095 | *8.60 x 10^-5^ | G | A | 0.35 | 1.06 x 10^-2^ |
| SD_SNP_000022773 | CM002085.1 | 5 | 40,129,189 | C | A | 0.4091 | *5.13 x 10^-5^ | C | A | 0.308 | 3.96 x 10^-3^ |
| SD_SNP_000022770 | CM002085.1 | 5 | 40,145,585 | A | G | 0.3706 | 1.03 x 10^-4^ | A | G | 0.409 | *9.08 x 10^-5^ |
| SD_SNP_000022766 | CM002085.1 | 5 | 40,158,838 | A | G | 0.3824 | 1.48 x 10^-4^ | A | G | 0.358 | *3.17 x 10^-6^ |
| SD_SNP_000026602 | CM002085.1 | 5 | 40,249,343 | A | G | 0.2818 | *9.19 x 10^-5^ | G | A | 0.312 | 6.52 x 10^-1^ |
| SD_SNP_000026599 | CM002085.1 | 5 | 40,269,392 | G | A | 0.0738 | *1.66 x 10^-8^ | G | A | 0.35 | 2.84 x 10^-4^ |
| SD_SNP_000019529 | CM002085.1 | 5 | 40,300,709 | A | G | 0.0751 | *1.51 x 10^-7^ | A | G | 0.323 | *6.13 x 10^-5^ |
| SD_SNP_000002370 | CM002085.1 | 5 | 40,396,733 | A | C | 0.0785 | *9.48 x 10^-6^ | A | C | 0.2 | *6.39 x 10^-5^ |
| SD_SNP_000016503 | CM002085.1 | 5 | 40,577,880 | G | A | 0.2658 | 2.35 x 10^-1^ | G | A | 0.223 | *8.77 x 10^-5^ |
| SD_SNP_000030214 | CM002085.1 | 5 | 40,587,726 | A | G | 0.2634 | 2.33 x 10^-1^ | A | G | 0.223 | *8.77 x 10^-5^ |
| SD_SNP_000030215 | CM002085.1 | 5 | 40,597,291 | A | G | 0.2723 | 4.38 x 10^-1^ | A | G | 0.217 | *5.51 x 10^-5^ |
| SD_SNP_000020190 | CM002085.1 | 5 | 40,902,353 | A | G | 0.0701 | *5.38 x 10^-5^ | A | G | 0.397 | 1.67 x 10^-3^ |
| SD_SNP_000020192 | CM002085.1 | 5 | 40,916,454 | G | A | 0.0731 | *7.76 x 10^-5^ | G | A | 0.419 | 2.73 x 10^-3^ |
| SD_SNP_000005964 | CM002085.1 | 5 | 41,036,282 | A | G | 0.0703 | 2.17 x 10^-4^ | A | G | 0.322 | *8.76 x 10^-6^ |
| SD_SNP_000009135 | CM002085.1 | 5 | 41,200,140 | A | G | 0.4002 | 2.29 x 10^-3^ | G | A | 0.148 | *5.51 x 10^-5^ |
| SD_SNP_000009134 | CM002085.1 | 5 | 41,203,008 | A | G | 0.0771 | 6.07 x 10^-3^ | A | G | 0.148 | *5.51 x 10^-5^ |
| SD_SNP_000009133 | CM002085.1 | 5 | 41,204,629 | G | A | 0.0772 | 6.07 x 10^-3^ | G | A | 0.148 | *5.51 x 10^-5^ |
| SD_SNP_000032691 | CM002086.1 | 6 | 41,545,028 | A | G | 0.4414 | *5.59 x 10^-5^ | A | G | 0.474 | 4.76 x 10^-1^ |
| SD_SNP_000032687 | CM002086.1 | 6 | 41,549,244 | A | G | 0.4414 | *5.59 x 10^-5^ | A | G | 0.474 | 4.76 x 10^-1^ |
| SD_SNP_000032683 | CM002086.1 | 6 | 41,557,789 | G | A | 0.4414 | *5.62 x 10^-5^ | G | A | 0.351 | 8.47 x 10^-1^ |
| SD_SNP_000038512 | CM002088.1 | 8 | 3,154,572 | G | A | 0.2639 | 2.45 x 10^-1^ | G | A | 0.095 | *2.15 x 10^-7^ |
| SD_SNP_000021743 | CM002088.1 | 8 | 5,393,101 | C | A | 0.2944 | 4.53 x 10^-1^ | C | A | 0.109 | *6.12 x 10^-6^ |
| SD_SNP_000043748 | CM002091.1 | 11 | 4,828,172 | A | G | 0.2455 | *7.67 x 10^-5^ | A | G | 0.041 | 1.00 x 10^+0^ |
| SD_SNP_000043747 | CM002091.1 | 11 | 4,831,923 | G | A | 0.2707 | *8.60 x 10^-5^ | G | A | 0.176 | 4.60 x 10^-1^ |
| SD_SNP_000043745 | CM002091.1 | 11 | 4,838,915 | G | A | 0.2706 | *8.19 x 10^-5^ | G | A | 0.176 | 4.60 x 10^-1^ |
| SD_SNP_000047737 | CM002091.1 | 11 | 5,165,520 | A | G | 0.2552 | *9.38 x 10^-6^ | G | - | 0.000 | 1.00 x 10^+0^ |
| SD_SNP_000037573 | CM002091.1 | 11 | 5,204,949 | A | G | 0.2457 | *3.25 x 10^-5^ | G | - | 0.000 | 1.00 x 10^+0^ |
| SD_SNP_000053510 | CM002091.1 | 11 | 5,255,710 | C | A | 0.2748 | *8.47 x 10^-5^ | C | A | 0.259 | 6.46 x 10^-1^ |
| SD_SNP_000031828 | CM002091.1 | 11 | 5,369,351 | A | C | 0.2545 | *1.24 x 10^-5^ | A | C | 0.39 | 8.83 x 10^-1^ |
| SD_SNP_000031829 | CM002091.1 | 11 | 5,372,237 | G | A | 0.2774 | *4.57 x 10^-6^ | G | A | 0.39 | 8.83 x 10^-1^ |
| SD_SNP_000046132 | CM002091.1 | 11 | 5,412,986 | A | G | 0.2519 | *2.97 x 10^-5^ | A | G | 0.049 | 1.00 x 10^+0^ |
| SD_SNP_000002502 | CM002091.1 | 11 | 5,420,279 | A | G | 0.2457 | *5.06 x 10^-6^ | A | G | 0.049 | 1.00 x 10^+0^ |
| SD_SNP_000002504 | CM002091.1 | 11 | 5,422,706 | G | A | 0.2521 | *3.46 x 10^-5^ | G | A | 0.051 | 8.15 x 10^-2^ |
| SD_SNP_000002507 | CM002091.1 | 11 | 5,436,885 | G | A | 0.2457 | *5.92 x 10^-6^ | G | A | 0.049 | 1.00 x 10^+0^ |
| SD_SNP_000002508 | CM002091.1 | 11 | 5,439,423 | G | A | 0.2522 | *3.44 x 10^-5^ | G | A | 0.213 | 5.22 x 10^-1^ |
| SD_SNP_000002510 | CM002091.1 | 11 | 5,442,401 | C | A | 0.2455 | *4.99 x 10^-6^ | C | A | 0.049 | 1.00 x 10^+0^ |

* The whole-genome significant values were determined at *p*≤10^-4^. The chromosome ID is based on the published physical map of oil palm with INSDC (International Nucleotide Sequence Database Collaboration) accessions.

**Supplementary Table 4. Summary of association results for SNPs in Deli x AVROS and Nigerian x AVROS.**

| SNP ID | INSDC | Chromosome | Position (bp) | Deli x AVROS | | | | Nigerian x AVROS | | | | * Genes in or near regions of association |
| --- | --- | --- | --- | --- | --- | --- | --- | --- | --- | --- | --- | --- |
|  |  |  |  | Major allele | Minor allele | MAF | *p* value | Major allele | Minor allele | MAF | *p* value |  |
| SD_SNP_000010418 | CM002085.1 | 5 | 34,828,628 | G | A | 0.380 | 2.391 x 10^-6^ | G | A | 0.247 | 2.45 x 10^-5^ | *AMC9* |
| SD_SNP_000019529 | CM002085.1 | 5 | 40,300,709 | A | G | 0.075 | 1.508 x 10^-7^ | A | G | 0.323 | 6.13 x 10^-5^ | *T12J13.9* |
| SD_SNP_000002370 | CM002085.1 | 5 | 40,396,733 | A | C | 0.078 | 9.483 x 10^-6^ | A | C | 0.200 | 6.39 x 10^-5^ | *PK* |

The association analysis based on compressed MLM model was performed in the Deli x AVROS group and the Nigerian x AVROS group. The whole-genome significance value was determined at *p*<10^-4^. ^*^The identification of a putative candidate gene was called when the SNP was within 4-Kb interval of either end. The chromosome ID is based on the published physical map of oil palm with INSDC (International Nucleotide Sequence Database Collaboration) accessions.
